# Policy Implications of the Southern and Central Africa International Center of Excellence for Malaria Research: Ten Years of Malaria Control Impact Assessments in Hypo-, Meso-, and Holoendemic Transmission Zones in Zambia and Zimbabwe

**DOI:** 10.4269/ajtmh.21-1288

**Published:** 2022-10-13

**Authors:** Amy Wesolowski, Matthew M. Ippolito, Mary E. Gebhardt, Ellen Ferriss, Jessica L. Schue, Tamaki Kobayashi, Mike Chaponda, Jean-Bertin Kabuya, Mbanga Muleba, Monicah Mburu, Japhet Matoba, Michael Musonda, Ben Katowa, Mukuma Lubinda, Harry Hamapumbu, Limonty Simubali, Twig Mudenda, Timothy M. Shields, Andre Hackman, Clive Shiff, Maureen Coetzee, Lizette L. Koekemoer, Shungu Munyati, Lovemore Gwanzura, Susan Mutambu, Jennifer C. Stevenson, Philip E. Thuma, Douglas E. Norris, Jeffrey A. Bailey, Jonathan J. Juliano, Gershom Chongwe, Modest Mulenga, Edgar Simulundu, Sungano Mharakurwa, Peter Agre, William J. Moss, for the Southern and Central Africa International Center of Excellence for Malaria Research

**Affiliations:** ^1^ Johns Hopkins Bloomberg School of Public Health, Baltimore, Maryland;; ^2^ Johns Hopkins University School of Medicine, Baltimore, Maryland;; ^3^ Tropical Diseases Research Centre, Ndola, Zambia;; ^4^ Macha Research Trust, Choma, Zambia;; ^5^ Wits Research Institute for Malaria, Faculty of Health Sciences, University of the Witwatersrand and National Institute for Communicable Diseases, Johannesburg, South Africa;; ^6^ Biomedical Research and Training Institute, Harare, Zimbabwe;; ^7^ University of Zimbabwe Faculty of Medicine and Health Sciences, Harare, Zimbabwe;; ^8^ Africa University, Mutare, Zimbabwe;; ^9^ Brown University, Providence, Rhode Island;; ^10^ University of North Carolina School of Medicine, Chapel Hill, North Carolina;; ^11^ Directorate of Research and Postgraduate Studies, Lusaka Apex Medical University, Lusaka, Zambia

## Abstract

The International Centers of Excellence for Malaria Research (ICEMR) were established by the National Institute of Allergy and Infectious Diseases more than a decade ago to provide multidisciplinary research support to malaria control programs worldwide, operating in endemic areas and contributing technology, expertise, and ultimately policy guidance for malaria control and elimination. The Southern and Central Africa ICEMR has conducted research across three main sites in Zambia and Zimbabwe that differ in ecology, entomology, transmission intensity, and control strategies. Scientific findings led to new policies and action by the national malaria control programs and their partners in the selection of methods, materials, timing, and locations of case management and vector control. Malaria risk maps and predictive models of case detection furnished by the ICEMR informed malaria elimination programming in southern Zambia, and time series analyses of entomological and parasitological data motivated several major changes to indoor residual spray campaigns in northern Zambia. Along the Zimbabwe–Mozambique border, temporal and geospatial data are currently informing investigations into a recent resurgence of malaria. Other ICEMR findings pertaining to parasite and mosquito genetics, human behavior, and clinical epidemiology have similarly yielded immediate and long-term policy implications at each of the sites, often with generalizable conclusions. The ICEMR programs thereby provide rigorous scientific investigations and analyses to national control and elimination programs, without which the impediments to malaria control and their potential solutions would remain understudied.

## INTRODUCTION

Despite substantial investments over the past 2 decades, many challenges remain to achieve malaria control and elimination along the continuum of transmission intensities in southern and central Africa.^1^ The current suite of tools, including vector control with indoor residual spraying (IRS) and insecticide treated nets (ITN), case management, and reactive case detection strategies, need to be implemented according to the local epidemiology, entomology, and ecology as well as optimally deployed at scale to minimize morbidity and mortality and achieve disease control and malaria elimination.^2^ Progress has stagnated at both ends of the transmission spectrum, from hypoendemic settings seeking to achieve and sustain malaria elimination to holoendemic settings aiming to reduce malaria morbidity and mortality.^1^ Tailored approaches are required that better account for the local epidemiology and vector bionomics, political environment, available resources, and heterogeneities in transmission intensities at fine spatial scales.^2^^,^^3^ In low-burden settings, such as southern Zambia, residual reservoirs of infection in the human population that are difficult to identify and eliminate can lead to persistent, low-level transmission.^4^^,^^5^ In some high-transmission settings, such as northern Zambia, malaria morbidity and mortality remain elevated despite intensive vector control (IRS, ITNs) and case management using rapid diagnostic tests (RDTs) and artemisinin-based combination therapy.^6^ Important considerations for countries such as Zambia and Zimbabwe are how should malaria control and elimination programs balance the substantial investments needed to achieve and sustain malaria elimination in low-transmission settings, where the cost per case averted is high, with investments in reducing the burden of malaria in moderate or high transmission zones.

**Figure 1.**
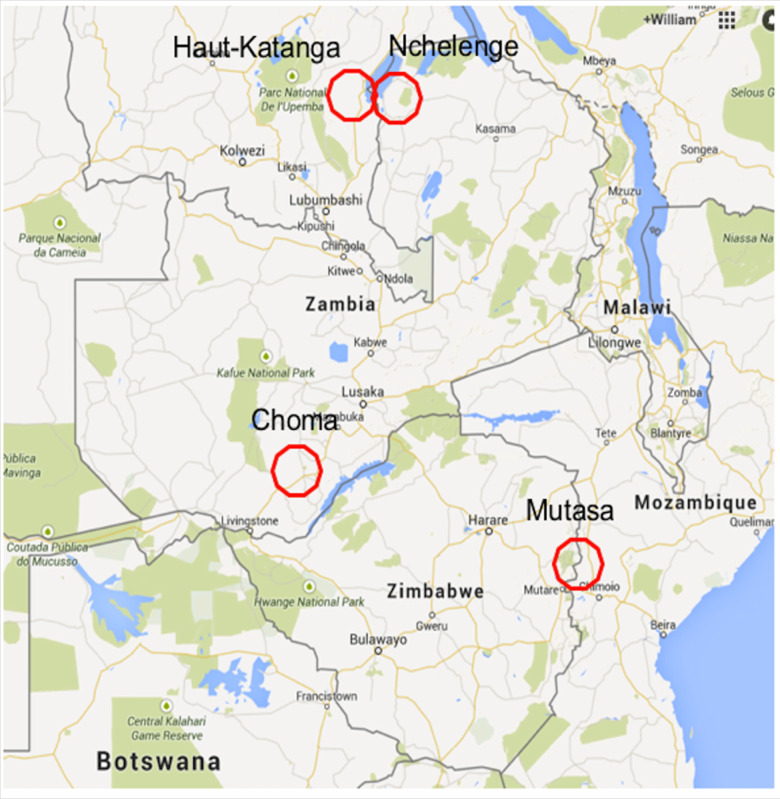
Southern and Central Africa International Centers of Excellence for Malaria Research study sites. This figure appears in color at www.ajtmh.org.

Many barriers remain to achieving malaria control and elimination in southern and central Africa, with additional insights needed to better implement and target existing as well as novel tools and strategies. We highlight policy implications of the integrated, interdisciplinary research conducted by the Southern and Central Africa International Center of Excellence for Malaria Research (ICEMR) in high and low transmission settings in Zambia and in a moderate transmission setting in eastern Zimbabwe along the border with Mozambique (Figure 1 and Table 1). Through studies of infections and clinical cases, vector dynamics, human and mosquito behavior, climate and environmental factors, and parasite and mosquito genetics, we sought to understand how these diverse factors drive transmission, impact the effectiveness of current interventions, and guide decisions and strategic plans on how more impactful and durable gains can be achieved.

**Table 1:** Policy implications of the Southern and Central Africa International Centers of Excellence for Malaria Research (ICEMR)

Transmission Setting	ICEMR Scientific Findings	Policy Implications
Low-transmission setting in Choma District, Zambia	Low sensitivity of RDTs for both active and passive surveillance Inability to detect hrp2 deletions in *Plasmodium falciparum* due to low parasitemia Most secondary cases are located in the index household Exophilic understudied vectors	Focal drug administration in index case household would achieve similar results to more costly reactive test-and-treat (Component D) Risk based reactive test-and-treat strategies, such as targeting households near streams and other high-risk environmental features could improve efficiency of RTAT Targeted IRS at index case households identified through passive case detection Expand entomological surveillance to identify and characterize underrecognized vectors Continued use of hrp2 RDTs Need for evaluation of outdoor vector control interventions More efficient and effective reactive case detection and focus investigations using a “1-3-7” focal intervention guided by a malaria mapping application Accurate and timely data reporting, analysis, and use at all levels
High-transmission setting in Nchelenge District, Zambia	Peak abundance of *An. funestus* in the dry season Documentation of persistent pyrethroid resistance in both major vectors Modest impact of IRS on malaria burden Modest impact of ITNs on malaria burden High case fatality among hospitalized children with severe malaria Excess malaria-attributable deaths due to stockouts of essential medical supplies	Increase IRS and ITN coverage throughout the district, in part through improved community mobilization and engagement Change timing of the IRS to end of the rainy season before *An. funestus* peaks Avoid pyrethroid-based insecticides for IRS and switch to PBO ITNs Continue to monitor insecticide resistance to more recently deployed insecticides Improve integrated community case management and make prereferral rectal or intramuscular artemisinin available at satellite health facilities Strengthen supply chains for antimalarial medications and blood products
Border transmission setting in Mutasa District, Zimbabwe	Pyrethroid resistance Reduction in malaria incidence after switch to primiphos-methyl IRS Persistent malaria burden, particularly along the border with Mozambique	Monitor insecticide resistance Avoid use of pyrethroids for IRS and switch to PBO ITNs Monitor cross-border malaria transmission Work with neighboring countries to harmonize and improve malaria control strategies and minimize imported malaria

IRS = indoor residual spraying; ITN = insecticide treated nets; PBO = piperonyl butoxide; RDT = rapid diagnostic tests; RTAT = reactive test-and-treat.

## PROGRAMMATIC IMPLICATIONS OF ICEMR FINDINGS FOR MALARIA ELIMINATION IN CHOMA DISTRICT, ZAMBIA

The burden of malaria has decreased dramatically in Southern Province, Zambia, over the past 2 decades, with gains in part sustained through improved case management with RDTs and artemisinin-based combination therapy, a reactive test-and-treat program, widespread distribution of ITNs, and selective use of IRS.^4^ Southern Province is targeted for malaria elimination by the Zambian National Malaria Elimination Center (NMEC). Thus, this area serves as a test case to assess the feasibility and effectiveness of interventions and strategies to achieve and sustain malaria elimination, including reactive test-and-treat and mass drug administration strategies.^7^^,^^8^ However, better understanding of the barriers to elimination—including the relative contributions of imported and local malaria, the significance of asymptomatic parasitemia, and the vectorial capacity of understudied, exophagic anophelines—is needed to develop effective, targeted strategies.

The Zambian National Malaria Elimination Strategic Plan for 2017–2021 had the following objectives in low-transmission settings: 1) interrupt malaria transmission; 2) report and respond to all confirmed cases to prevent continued transmission; 3) determine the underlying causes of residual transmission; and, if successful, 4) maintain and document malaria elimination.^9^ This is operationalized through Component D of the overall strategy, which involves detecting and investigating individual cases in index and neighboring households to interrupt transmission. Component D is implemented in low transmission areas, such as Southern Province, where parasite prevalence is approximately 1% and an average of 10 or fewer malaria cases present to a health facility per week. The Zambian NMEC implemented Component D in Southern Province starting in 2013 through a reactive test-and-treat program in which symptomatic individuals with a positive RDT are followed up at their home within 1 week of diagnosis and residents of the index case household and residents of neighboring households within a 140-meter radius of the index case household are tested with an RDT and treated if positive within 1 week.

Since 2010, the Southern and Central Africa ICEMR has conducted epidemiological and entomological studies to better understand how to achieve and sustain malaria elimination in Choma District, Southern Province, Zambia. These studies have been conducted by Macha Research Trust in the catchment area of Macha Hospital, an area of rural subsistence farming with a single rainy season from November to April during which malaria transmission peaks.^7^^,^^10^ Parasite prevalence, measured by active surveillance, declined substantially over the past decade to approximately 1%. Using cross-sectional and longitudinal cohort study designs, the Southern and Central Africa ICEMR has sought to understand the temporal and spatial dynamics of malaria transmission, the responsible vectors, and the characteristics of transmission foci (i.e., hot spots or hot populations) that should be targeted to reduce transmission and achieve elimination.^4^^,^^5^^,^^7^^,^^10^^,^^11^ Such studies are critically important but inherently challenging because precise measurements in settings with low malaria incidence and low parasite prevalence require large sample sizes to capture sufficient outcomes, and low levels of parasitemia in infected individuals make high-resolution parasite genotyping challenging.

The underlying assumption of reactive test-and-treat strategies is that they can identify malaria hotspots and interrupt transmission foci. This strategy leverages the temporal and spatial clustering of malaria transmission while minimizing exposure to antimalarials, in contrast to mass drug administration strategies.^12^ Reactive strategies become increasingly important as transmission declines because the impact of one missed case on onward transmission is greater in such settings. However, the Southern and Central Africa ICEMR found that RDTs were insufficiently sensitive to identify individuals with low-level parasitemia and most reactively detected infected individuals resided within the index case household and not in neighboring households.^7^ More efficient strategies include focal drug administration to all residents within the index household and, in the future, point-of-contact molecular diagnostic tests. In follow-up studies to explore temporal aspects of transmission by including additional 30- and 90-day study visits after the initial reactive-test and treat event, parasite prevalence remained low but persisted as detected by quantitative polymerase chain reaction (qPCR).^4^ Parasite genotyping showed that reactive test-and-treat succeeded in identifying local transmission chains,^13^ but the operational challenges of following up all index cases and tracking all household members hindered its effectiveness.^10^ These findings suggest that reactive test-and-treat strategies based on RDTs are ineffective and inefficient, as the low sensitivity of RDTs results in a large proportion of missed infections. Furthermore, investigating neighboring households may not result in much additional benefit because most parasitemic individuals were identified within the index case household.^7^ More targeted reactive test-and-treat using environmental risk factors such as proximity to potential vector breeding sites (e.g., streams) could improve efficiency.^11^ However, a strategy of focal drug administration without RDT testing in the index household, and without secondary household screening, would achieve close to the same impact as the current reactive test-and-treat strategy at lower cost and higher efficiency.^7^

Vector control strategies in Choma District consist largely of ITN distributions, with occasional targeted IRS. Although indoor vector counts were low in Southern and Central Africa ICEMR collections, more anophelines were captured inside households with qPCR-positive cases, making index case households more appropriate candidates for indoor entomological interventions like IRS. However, Southern and Central Africa ICEMR studies suggest that transmission is also occurring outdoors by understudied vectors such as *An. squamosus* and *An. rufipes*, underscoring the necessity for further evaluation of vector control interventions that are effective outdoors, including attractive toxic sugar baits and spatial repellents.^14^

Countries that recently achieved malaria elimination implemented individual-case-based surveillance based on reactive case detection but further deployed “focus investigations,” which routinely surveils focal areas of transmission and manages targeted response efforts.^15^ China and neighboring southeast Asian countries have been successful in operationalizing focus investigation through a strategy called “1-3-7” that institutionalizes response times and specific decision support protocols for case classification, focus classification, and intervention response at 1, 3, and 7 days, respectively, after identifying the index case.^16^ Deploying such a strategy could enhance reactive case detection and promote local management of transmission through focus investigation. However, this strategy has not been deployed or evaluated in sub-Saharan Africa. The Southern and Central Africa ICEMR is evaluating a “1-3-7” strategy in Choma District in 2022 using a mobile application developed by the Southern and Central Africa ICEMR to track cases in space and time at the level of health zones in near real-time.

Studies of low-level malaria transmission in Choma District by the Southern and Central Africa ICEMR highlight the limitations of reactive test-and-treat and indoor vector control strategies in achieving and sustaining malaria elimination. Focal drug administration in the index case household may be a more efficient strategy than reactive test-and-treat in neighboring households.^7^ Outdoor vector control interventions are needed in Choma District in addition to indoor-directed measures because of the presence of several exophilic vectors.^17^ Sustainable malaria surveillance, with detailed and timely data collection, analyses, and use for decision-making, that leads to focal responses should be integrated into the primary health care system and can leverage user-friendly mobile phone applications. Such a system, developed by the Southern and Central Africa ICEMR, can empower health workers at the level of individual health facilities to make decisions and respond to cases in a timely manner.

## PROGRAMMATIC IMPLICATIONS OF ICEMR FINDINGS FOR MALARIA CONTROL IN NCHELENGE DISTRICT, ZAMBIA

In contrast to southern Zambia, Nchelenge District, Luapula Province in northern Zambia has high perennial malaria transmission with among the highest incidence rates and case fatality ratios in the country.^18^ In such holoendemic areas, policies focus on bringing to scale interventions to prevent infection and reduce morbidity and mortality, including enhancing and optimizing vector control and case management.

Over the past decade, IRS has been conducted annually before the rainy season in increasingly larger spray areas, and the insecticide has changed three times: from bendiocarb (Ficam^®^) in 2012 to pirimiphos-methyl (Actellic^®^ 300CS) in 2014, to deltamethrin-clothianidin (Fludora^®^ Fusion) in 2019, and to clothianidin (SumiShield^®^) in 2021.^6^ However, targeted IRS before the onset of the rainy season has been insufficient to reduce malaria transmission and burden in Nchelenge District.^6^^,^^19^ The Southern and Central Africa ICEMR, in partnership with the Tropical Diseases Research Centre, identified several possible causes for the minimal impact of IRS on parasite prevalence: 1) low district-level coverage; 2) potential exophilic behavior of *An. funestus*; 3) frequent movement of individuals between sprayed, lower risk lakeside areas and unsprayed, higher risk inland areas; and, most important, 4) persistent year-round malaria transmission with *An. funestus* abundance peaking during the dry season.^6^^,^^19^^–^^22^ In holoendemic settings such as Nchelenge District with distinctive local entomology and ecology, an IRS strategy tailored to the local context is necessary. Changes to the timing and frequency of IRS spraying, in addition to increasing household coverage of IRS to the recommended 80% minimum or higher,^23^ will be needed to achieve malaria control, specifically the need to conduct IRS twice a year or, alternatively, at the end rather than the beginning of the rainy season. ITNs have continued to provide protection despite pyrethroid resistance in both *An. funestus* and *An. gambiae*,^24^ supporting their role as an important vector control strategy in Nchelenge District. Given the high levels of pyrethroid resistance in both vectors, nonpyrethroid insecticides for IRS should continue to be used in combination with ITNs. The detection of outdoor biting vectors warrants the generation of local data to investigate interventions that target exophagic vectors.

Malaria accounts for more than one-third of pediatric hospitalizations and nearly half of inpatient pediatric deaths in Nchelenge District.^25^ Thus, policies that succeed in controlling malaria will translate to a substantial number of lives saved. The presence of the Southern and Central Africa ICEMR in Nchelenge District motivated the Zambian NMEC in partnership with the U.S. President’s Malaria Initiative (PMI) to select the district as a test site for intensive malaria control interventions for Zambia’s high malaria transmission regions. In 2018, Southern and Central Africa ICEMR findings regarding risk factors associated with severe malaria led in part to the policy decision by the NMEC and PMI to train and deploy more than 300 new community health workers in Nchelenge District as part of an integrated community case management (iCCM) program. The intent was to increase access to care and promote early diagnosis and referral of malaria based on a Southern and Central Africa ICEMR analysis of hospitalized children with malaria that identified distance from a patient’s home village to the hospital as the strongest predictor of survival.^25^ Impact assessments of the iCCM program are ongoing, but preliminary findings suggest that the program succeeded in its first year in referring children with severe malaria in the community who otherwise may have gone undertreated, including those from more remote rural villages.^26^^,^^27^

Southern and Central Africa ICEMR surveillance activities of severe malaria also captured the extent to which supply chain interruptions of intravenous artesunate and whole blood—the latter used to treat children with severe malarial anemia, the most common form of severe malaria in high-burden areas—led to preventable deaths.^25^ Excess malaria deaths due to stockouts of essential medical supplies are not typically accounted for by national malaria control programs or the WHO in their estimates of malaria deaths and case fatality. After the Southern and Central Africa ICEMR disseminated these findings in Zambia, PMI recently planned investments in infrastructure to support blood banks in Zambia while the NMEC continues to optimize supply chains with Medical Stores Ltd. and other public–private partnerships.

Through rigorous analyses accounting for environmental factors, weather patterns, and other potential confounding factors, the Southern and Central Africa ICEMR documented only modest impacts of IRS, ITNs, and iCCM on malaria burden in Nchelenge District. The first goal in Nchelenge District should be to reduce severe malaria morbidity and mortality. This will require early diagnosis and treatment in the community, including prereferral rectal artesunate or equivalent, prompt transfer to the hospital, and ensuring against stockouts of artesunate, blood, and other essential medical resources. Several factors likely explain why vector control interventions had minimal impact, most notably the suboptimal timing of IRS at the beginning of the rainy season rather than during the dry season when the most abundant vector, *An. funestus*, peaks.^19^ These ICEMR findings have led to the NMEC, PMI, and their partners to pilot a change in the timing of the annual IRS campaign to the end of the rainy season in 2022, and the ICEMR will evaluate the impact of this change in IRS timing. However, twice-yearly IRS may be needed in holoendemic settings such as Nchelenge District to have a substantial impact on the burden of malaria, and the appropriate level of resources will be needed to support such a strategy. Ensuring high coverage of IRS within targeted spray areas, as well as expanding IRS to most of the district, will also be necessary to achieve the indirect community-level protection conferred by IRS. Despite recommendations from the WHO that households receive either IRS or ITNs, households in high-transmission settings with pyrethroid resistance, such Nchelenge District, require both interventions.^2^^,^^3^ The prevalence of pyrethroid resistance also supports the use of piperonyl butoxide ITNs rather than pyrethroid-only ITNs.^28^ Engagement with partners beyond the health sector, such as environmental and housing ministries, will be needed to promote improvements in household structure that reduce contact with malaria vectors and provide better surfaces for effective IRS. Improved housing and appropriate environmental management have long been recognized as important for malaria control and need to be part of the solution in holoendemic settings like Nchelenge District.^29^^,^^30^ Finally, engagement with the Expanded Program on Immunization will be needed to optimize deployment of the RTS,S malaria vaccine when Gavi funding becomes available to Zambia.

## PROGRAMMATIC IMPLICATIONS OF ICEMR FINDINGS FOR MALARIA CONTROL AND ELIMINATION IN MUTASA DISTRICT, ZIMBABWE

As Zimbabwe moves toward malaria elimination, malaria transmission across or along international borders becomes increasingly salient. In 2016, 82% of Zimbabwe’s malaria cases were recorded in the country’s three eastern border provinces abutting Mozambique.^31^ The southernmost of these provinces, Manicaland Province, accounted for 39% of cases nationally. Zimbabwe has prioritized strengthening malaria control at the border through case management for mobile and migrant populations, targeted social and behavior change messaging, and prevention and treatment services.^32^ In addition, the Elimination 8 Regional Initiative (E8) to eliminate malaria in southern Africa has identified reduction of cross-border malaria as a priority.^33^ As malaria transmission declines in Zimbabwe, maintaining accessible healthcare and high coverage of vector control in at-risk areas and preventing importation become increasingly necessary.

The Southern and Central Africa ICEMR, in partnership with the Biomedical Research and Training Institute, Africa University, and the National Health Research Institute, conducted epidemiological and entomological studies of malaria in Mutasa District, Manicaland Province in eastern Zimbabwe on the border with Mozambique for almost a decade. The Southern and Central Africa ICEMR documented pyrethroid resistance in *An. funestus*, leading to a change in the insecticide used for IRS to pirimiphos-methyl (Actellic™).^28^ The Southern and Central Africa ICEMR then documented a substantial reduction in the burden of malaria the first year after IRS with pirimiphos-methyl but subsequently identified the persistent and increasing malaria burden, particularly along the border with Mozambique.^34^ Passive primary healthcare–based surveillance and community-based active surveillance conducted by the Southern and Central Africa ICEMR demonstrated a risk gradient running from the Eastern Highlands to Honde Valley that mirrors local variation in elevation, ecology, and human population movement between Zimbabwe and Mozambique, where malaria transmission is more intense.^35^ Although clinical malaria is associated with lower elevation and higher rainfall and nighttime temperatures, environmental characteristics do not fully explain the increased parasite prevalence closer to the border, suggesting travel between countries is contributing to transmission.^35^ The Southern and Central Africa ICEMR is investigating ways to quantify the contribution of cross-border malaria transmission as well as mitigation strategies.

Although malaria control in Mutasa District is not specifically targeted to the border population, traditional strategies, including case management and vector control, have been instrumental. Drug and insecticide resistance monitoring by the Southern and Central Africa ICEMR have facilitated more effective programming.^28^^,^^36^^,^^37^ In 2003, after the development of chloroquine resistance, Zimbabwe discontinued chloroquine monotherapy. The Southern and Central Africa ICEMR monitoring in Mutasa District demonstrated reversion to chloroquine-susceptible *Plasmodium falciparum* by 2018.^37^ Insecticide resistance has similarly challenged control efforts. After demonstration of pyrethroid resistance by the Southern and Central Africa ICEMR in 2014, Mutasa District transitioned to pirimiphos-methyl for IRS, effecting a lasting albeit partial reduction in clinical cases^34^ up until recently when, after a switch to dichloro-diphenyl-trichloroethane (DDT) and a handover of the IRS program from PMI to the NMCP in 2018, reported cases resurged and then plummeted during the pandemic (unpublished data). Despite pyrethroid resistance, ITNs have been associated with decreased risk of malaria,^38^^,^^39^ although restoration of local vector susceptibility to deltamethrin in the presence of PBO presents an opportunity for improved outcomes with pyrethroid-PBO nets.

Although environmental risk factors for malaria have been identified and ongoing parasite importation from Mozambique is supported by the Southern and Central Africa ICEMR findings, malaria transmission at the border is still incompletely understood. Future research will seek to clarify where and when individuals are infected to optimize the geographic deployment of control interventions. Continued case management and high coverage of vector control interventions will be necessary to sustain reductions in malaria transmission and respond to the threat of resurgence. New strategies to prevent parasite importation will also be needed. The E8’s support of regional coordination and policy harmonization in border regions will enable better access to healthcare at the border and facilitate synchronized cross-border malaria control programming.^33^ Additional opportunities for malaria control in eastern Zimbabwe include screening and treating individuals at formal and informal border crossings and through public–private partnerships with institutions serving mobile and migrant communities.

## CONCLUSION

By providing insights into the limitations of current malaria control and elimination interventions, investigations by the Southern and Central Africa ICEMR have helped inform policy across three transmission settings in two countries. However, multiple impediments exist to translate scientific findings into policy, including the need to communicate research findings effectively to policy makers and subsequently to have the motivation and resources to adapt and implement the recommendations at scale. These barriers are not trivial and require continued engagement and commitment. The Southern and Central Africa ICEMR participates in several technical advisory committees that support the national malaria elimination and control programs, providing opportunities for research findings to be presented and discussed. Some recommendations, such as biannual IRS in Nchelenge District, Zambia, are not currently financially feasible. On the other hand, the community in Nchelenge District is willing to consider a change in the timing of IRS to the end of the rainy season, as demonstrated in a recent pilot project.

Finally, the value of the Southern and Central Africa ICEMR in informing strategies, policies, and programs are an outcome of two critical features of the program: 1) the multidisciplinary approach that combines concurrent epidemiological, entomological, geospatial, and parasite and vector genetic studies and 2) the duration of the project that allows for the generation of long-term datasets to account for secular changes in weather patterns, vector abundance, and malaria transmission that would otherwise confound interpretations. These Southern and Central Africa ICEMR studies lay the foundation for the optimal deployment of existing and novel interventions, including malaria vaccines, seasonal malaria chemoprevention, highly sensitive rapid diagnostic tests, outdoor vector control, and genetically or microbially modified mosquitoes, thus helping pave the way for the future of malaria control.
